# The Insurance Bill

**Published:** 1911-06-03

**Authors:** B. Wagstaff

**Affiliations:** Secretary Royal Portsmouth Hospital. Portsmouth


### INSTITUTIONAL LETTER-BOX.

### THE INSURANCE BILL.

*To the Editor of* The Hospital.

Dear Sir,—The excellent article in your issue of the 27th inst., written by Mr. Walter Carnt, appears to me clearly to indicate the probable effect of the National Insurance Bill upon the voluntary hospitals. All his remarks upon the subject, it seems to me, apply to the Royal Portsmouth Hospital.—Yours faithfully,

B. Wagstaff,
Secretary Royal Portsmouth Hospital.

Portsmouth,
May 29.

